# Predicting the potential implications of perch (*Perca fluviatilis*) introductions to a biodiversity-rich lake using stable isotope analysis

**DOI:** 10.1038/s41598-023-44865-2

**Published:** 2023-10-17

**Authors:** Ali Serhan Tarkan, Phillip J. Haubrock, Sadi Aksu, Oğuzcan Mol, Paride Balzani, Özgür Emiroğlu, Esengül Köse, Irmak Kurtul, Sercan Başkurt, Emre Çınar, Pinar Oztopcu-Vatan

**Affiliations:** 1 Department of Basic Sciences, Faculty of Fisheries, Muğla Sıtkı Koçman University, Menteşe, Muğla Turkey; 2 Department of Life and Environmental Sciences, Faculty of Science and Technology, Bournemouth University, Poole, Dorset UK; 3 Department of Ecology and Vertebrate Zoology, Faculty of Biology and Environmental Protection, University of Łodz, Łodz, Poland; 4 Department of River Ecology and Conservation, Senckenberg Research Institute and Natural History Museum Frankfurt, Gelnhausen, Germany; 5 Faculty of Fisheries and Protection of Waters, South Bohemian Research Centre of Aquaculture and Biodiversity of Hydrocenoses, University of South Bohemia in České Budějovice, Zátiší 728/II, 389 25 Vodňany, Czech Republic; 6 CAMB, Center for Applied Mathematics and Bioinformatics, Gulf University for Science and Technology, Al-Abdullah, Kuwait; 7 Vocational School of Health Services, Eskişehir Osmangazi University, Eskişehir, Turkey; 8 Department of Biology, Faculty of Science, Eskişehir Osmangazi University, Eskişehir, Turkey; 9 Department of Environmental Protection, Technologies, Eskişehir Osmangazi University, Eskişehir, Turkey; 10 Marine and Inland Waters Sciences and Technology Department, Faculty of Fisheries, Ege University, İzmir, Turkey

**Keywords:** Animal migration, Behavioural ecology, Community ecology, Ecological modelling, Ecosystem ecology, Invasive species

## Abstract

Biological invasions, particularly of fish species, significantly threaten aquatic ecosystems. Among these invaders, the introduction of the European perch (*Perca fluviatilis*) can have particularly detrimental effects on native communities, affecting both ecosystem functioning and human well-being. In this study, carbon and nitrogen stable isotope analysis was employed, using perch originating from five different ecosystems, to model the effects of their hypothetical introduction into İznik Lake, an economically and ecologically important, biodiversity-rich lake in northern Turkey, to ultimately assess their potential predation impact and competition with native predators. The results revealed that if perch were introduced to the community, they would – considering gape size limitations – primarily prey upon *Vimba vimba* and *Rutilus rutilus*, indicating a significant feeding pressure on these species. Furthermore, the study identified a potential overlap and competition for resources between commonly mesopredator perch and the European catfish *Silurus glanis*, the current top predator in the ecosystem. Both species would occupy top predatory positions, emphasizing the potential disruption of predator–prey dynamics. Our findings underscore the potential ecological repercussions of perch invasions. The selective predation on *V. vimba* and *R. rutilus,* with the latter being consumed to a lesser extent by perch, could lead to cascading effects throughout the food web, altering the community structure, and ecosystem dynamics. Additionally, the competition between perch and *S. glanis *raises concerns about effects on the stability and functioning of the fish community. These results highlight the need for proactive management strategies to mitigate the risk of perch introductions. Strict regulations on the movement and introduction of invasive species, along with comprehensive monitoring, are crucial for preserving native communities and maintaining the ecological integrity of freshwater ecosystems. Our study demonstrates the potential predation impact of perch on vulnerable fish species and the competition with the established apex predator, emphasizing the importance of considering the ecological consequences of perch invasions and informing management decisions to ensure the conservation and sustainability of aquatic ecosystems.

## Introduction

Biological invasions, specifically those occurring in freshwater ecosystems^1^, have emerged as a significant environmental concern in recent years^2^. Invasive species, i.e. organisms introduced into an ecosystem outside their natural range, often because of human activities where they establish self-sustaining populations and spread, have the potential to disrupt native ecosystems, leading to detrimental ecological and economic consequences^3–5^. These species often possess competitive advantages, allowing them to outcompete native species for resources^6^. Freshwater invasions are distinctive within the broader context of biological invasions due to the insular nature of lake ecosystems and high connectivity of rivers and streams. This susceptibility to invasive species' impacts presents a challenge for invasion risk and impact assessments^7^.

Due to its natural biogeographical frontiers^8^, freshwater ecosystems in Turkey hold significant ecological and economic importance, harboring diverse species and supporting numerous human activities^9^. However, these ecosystems face escalating threats from biological invasions, which pose a serious risk to their integrity and functionality^10^. This is mainly because of the large transboundary river systems running through the country that increase the risk of non-native fish introductions from both Asia and Europe, a hotspot of freshwater fish diversity and endemism^8^. To date, 384 freshwater fish species have been recorded from Turkey, of which 208 (54%) are endemic and 15 (4%) non-native^11^. Often overlooked, nationally translocated species—those that are moved from one region within a country where they are native to another region where they are not—can pose a significant risk to native species and ecosystems^12–15^. Translocated fish are of potential concern, as many demonstrate a high invasiveness in Europe (e.g. Gobiidae)^12^. Despite being particularly common in some countries such as Turkey^10^, translocated species are probably the least regulated among fish movements, whether they are for fisheries, aquaculture or ornamental (i.e. aquaria or garden ponds) purposes, with secondary spread being common in most cases^16,17^. Nonetheless, frequent translocations occur within Anatolia, involving *Alburnus alburnus*, *Garra rufa*, *Rutilus rutilus*, *Esox lucius* and, recently, the European perch *Perca fluviatilis*^9^.

Fish invasions can have wide-ranging and often severe impacts on native communities and ecosystem functioning^18–20^. Predation and competition are among the most common effects associated with fish invasions^21,22^. Predatory fish can alter prey populations, leading to cascading effects throughout the food web^23^. Similarly, competition between invasive and native species for limited resources can disrupt ecological balances, potentially driving native species to local extinctions^7^. The European perch, which is widely distributed throughout Eurasia, has been introduced to a few small reservoirs in Portugal mainland and river systems in Portuguese Azores islands, Catalonian river basins including the Ebro delta in Spain, central and southern Italy, Lake Skadar (Montenegro, Albania), Amur (Siberia), Australia and – among other regions – South Africa, where it has been recognized as an invasive species, posing significant threats to native biodiversity^24,25^. Its aggressive feeding behavior often results in the extirpation of native fish species^26^, as for instance anglers’ observations indicate that the aggressiveness of the species is more pronounced in artificial ecosystems (i.e. reservoirs) where it has been translocated compared to its native populations in natural lakes. Concomitantly, when introduced, perch may become a preferred prey species for the native European catfish *Silurus glanis*^27,28^, affecting currently established trophic networks and predator prey dynamics^29^.

To better understand the ecological dynamics of invasive fish species and their impacts on native communities, stable isotope analysis has become a valuable tool^30–32^, as nitrogen and carbon stable isotopes provide unique signatures that can be used to trace energy flow within food webs and elucidate trophic interactions, allowing researchers to investigate the feeding habits, trophic positions, and dietary preferences of invasive species, as well as their potential impacts on native communities^30^. Understanding trophic positions, which denote an organism's place in the food web, ranging from primary producers to consumers and higher-level predators, is crucial in this context^33^. This information is provided by the nitrogen stable isotope signature of a certain consumer, which is enriched by a predictable factor (the trophic discrimination factor) compared to its prey^34^, providing essential insights into the flow of energy and nutrients within an ecosystem. Together with carbon stable isotope values, which inform about the carbon pathway of the nutrient and biomass flow^33^ and allow to define an isotopic niche^35^, this comprehensive understanding sheds light on the potential impacts of species reintroduction and invasion events on ecological dynamics^20,30^. In recent years, stable isotope analyses have been applied to predict the ecological consequences of species reintroduction and invasion events^20^. Notably, Balzani and Haubrock^35^ developed a conceptual framework to use stable isotopes data to approximate potential impacts of biological invasions on trophic webs. This approach has concomitantly proven valuable in assessing the potential ecological repercussions of reintroducing a species that had previously gone extinct and its subsequent effects on the lake community^20^.

Understanding the potential impacts of fish invasions on often highly anthropogenically affected lake ecosystems is more important than ever, as they are home to often unique species communities and in many cases, endemic species, while forming the basis of effective management and conservation efforts^36,37^. Hence, in the present study, we adopted the approach proposed by Balzani and Haubrock^35^ to evaluate the potential impacts of perch introductions from two types of source populations (reservoirs and natural lakes) on native communities, using as a model site the ﻿İznik Lake in Turkey. Specifically, we investigated the predation on lower trophic levels and the competition with other species occupying similar trophic positions. Considering the ethological differences between the source populations of perch, we hypothesized that the theoretical impacts would vary, resulting in differences in the predation impact and competition with other predators. The findings from our study will have important implications for ecosystem managers, stakeholders, and policymakers, highlighting the significance of proactive measures in mitigating biological invasions before they occur. By understanding the potential impacts of invasive species introductions, we can develop targeted strategies for invasive species management and prioritize the preservation of native communities and the ecological integrity of freshwater ecosystems.

## Results

**Figure 1:**
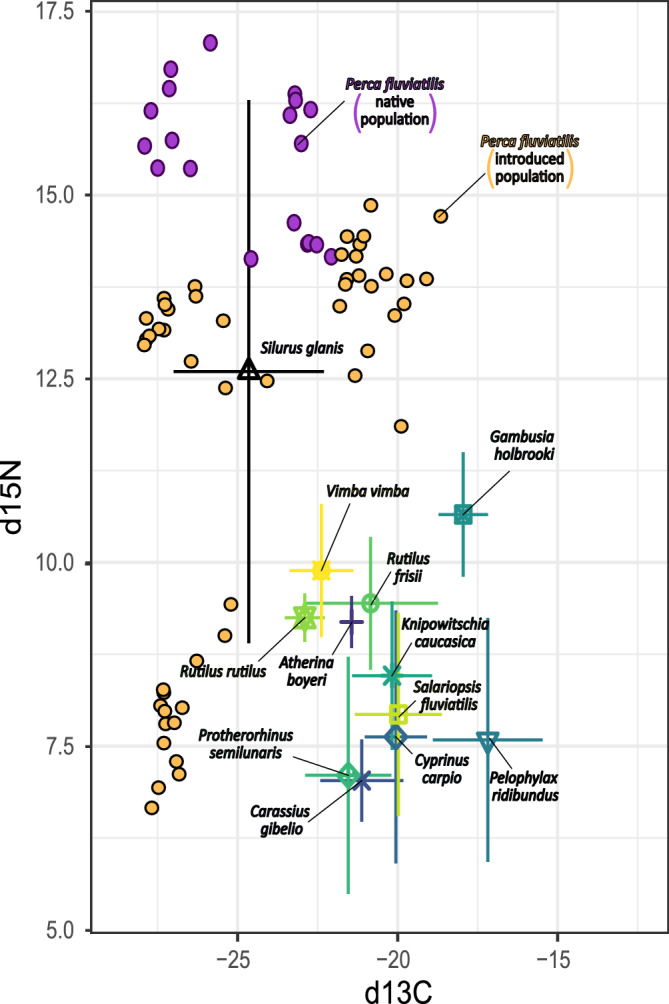
Carbon (δ^13^C) and nitrogen (δ^15^N) stable isotopes biplot of the ﻿İznik Lake fish community, with potential prey species for native and possibly introduced *Perca fluvialitis*.

In ﻿İznik Lake, *Silurus glanis* was considered the only apex predator, with only *Gambusia holbrooki* distinguishing from the otherwise overlapping fish species (Fig. 1). δ^15^N values were on average higher in lakes (15.5‰) than in reservoirs (11.8‰), while the variance in both δ^13^C and δ^15^N were wider in reservoirs (Table 1). Projecting the stable isotope signatures of the two groups of *Perca fluviatilis* indicated a strong potential overlap with the *S. glanis* population (Fig. 1). Although not yet occurring in syntopy, the trophic position of the fish species in ﻿İznik Lake, indicated that potentially introduced *P. fluviatilis* from both lakes (TP = 5.39) and reservoirs (TP = 4.28), as well as *S. glanis* (TP = 4.46) would be occupying top predator positions. The TP of other fish species ranged from 3.95 (*G. holbrooki*) and 3.60 (*Vimba vimba*) to as low as 2.82 (*Carassius gibelio*).

**Table 1:** Average total length (TL), weight (W), δ^15^N and δ^13^C of native and translocated *Perca fluviatilis* from lakes and reservoirs.

System	Status	Site	δ^15^N [‰]	δ^13^C [‰]	TL [mm]	W [g]
Lake	Native	Gala	15.8	− 27.5	153.0	59.9
Lake	Native	Sigirci	15.2	− 23.6	172.6	111.8
Mean			15.5	− 25.4	163.3	87.2
Reservoir	Translocated	Bayat	7.9	− 28.5	158.0	44.8
Reservoir	Translocated	Ozburun	13.2	− 28.4	169.3	61.4
Reservoir	Translocated	Seyitler	13.8	− 21.9	229.3	174.2
Mean			11.8	− 25.8	189.9	101.5
Mean (overall)			12.9	− 25.7	182.6	97.6

### Potential dietary impact

**Figure 2:**
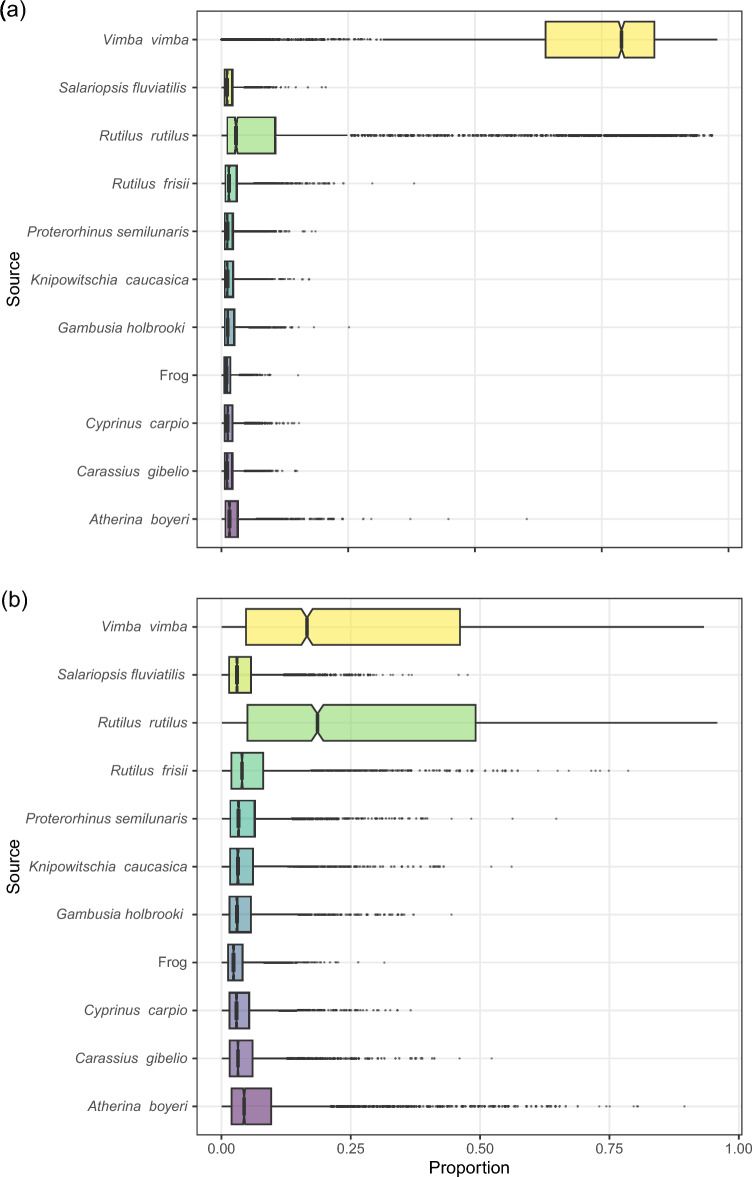
Mixing models results for (**a**) introduced perch vs (**b**) native perch.

Stable isotope mixing models suggested that if introduced to ﻿İznik, both *P. fluviatilis* from reservoirs where it was previously introduced to (Fig. 2a) and lakes where it was native to (Fig. 2b) would result in the primary predation of *Vimba vimba* and *Rutilus rutilus*, albite the latter appearing to a lesser degree in *P. fluviatilis* from reservoirs. All other species were indicated as unlikely to be consumed by *P. fluviatilis* in both cases. However, running stable isotopes mixing models separately for each source population of perch indicated variations, with predated species, such as *Proterorhinus semilunaris*, *C. gibelio*, and *Atherina boyeri*, may be targeted by perch predation, albeit to a lesser degree (Fig. S1). Our predictive models also suggest that *C. gibelio*, the most common invasive fish species in Turkish freshwaters, would be preyed upon by translocated perch in Bayat reservoir (Fig. S2).

### Niche overlap

**Figure 3:**
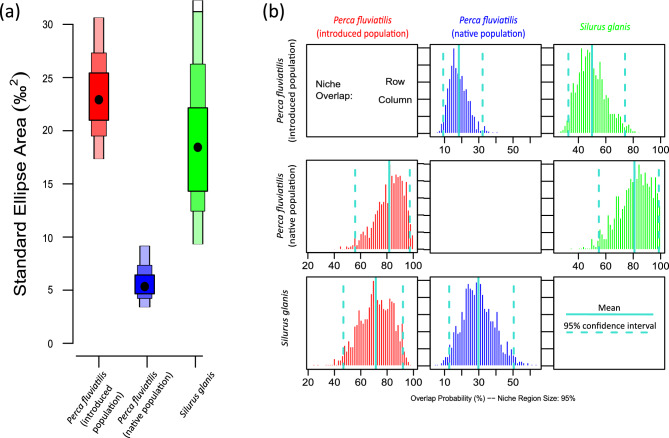
A posteriori distributions for the Bayesian standard ellipse areas (SEAb) (**a**) and the niche overlap based on the 95% confidence interval (SEAb) (**b**) for native *Perca fluviatilis* from lakes, introduced *P. fluviatilis* from reservoirs, and *Silurus glanis* native to ﻿İznik Lake.

With regard to the possibility of competition between introduced perch and other predators (i.e. *S. glanis*) in ﻿İznik Lake, we identified considerable overlap potential if *P. fluviatilis* was to be introduced. We found that, following the theoretic introduction of *P. fluviatilis*, *S. glanis* would overlap in terms of the 95% Bayesian standard ellipse area (SEAb) with both introduced *P. fluviatilis* from reservoirs (27.2% overlap) and native *P. fluviatilis* from lakes (29.4% overlap). *Silurus glanis* furthermore expressed the widest SEAb, followed by introduced *P. fluviatilis* from reservoirs and native *P. fluvialits* from lakes, with the latter expressing a comparably small and condensed niche (Fig. 3a). Considering SEAb, the highest potential of directional overlap was between native and introduced *P. fluviatilis* (82.5%), followed by the likelihood of introduced *P. fluviatilis* overlapping with native *S. glanis* (81.0%). Considering the 40% corrected standard ellipse areas (SEAc), the overlap potential was much lower. *Perca fluviatilis* from lakes and reservoirs both had a considerable potential to overlap with *S. glanis* (18.1 to 21.9%). The probability of *S. glanis* overlapping ranged from 29.9% (*P. fluviatilis* from reservoirs) to 8.6% (*P. fluviatilis* from lakes; Fig. 3b; Table 2).

**Table 2:** Directional pairwise probability of niche overlap (i.e. the probability of an individual of species A to fall within the niche of species B) for the Bayesian standard ellipse areas (SEAb, 95% confidence interval) and corrected standard ellipse areas (SEAc, 40% confidence interval) between the established native *Silurus glanis* and the two potential populations of *Perca fluviatilis*.

		Species B		
		*Perca fluviatilis* (introduced)	*Perca fluviatilis* (native)	*Silurus glanis*
SEAb				
Species A	*Perca fluviatilis* (introduced)	NA	18.46	49.62
	*Perca fluviatilis* (native)	82.50	NA	81.00
	*Silurus glanis*	70.98	29.71	NA
SEAc				
Species A	*Perca fluviatilis* (introduced)	NA	3.30	18.06
	*Perca fluviatilis* (native)	8.37	NA	21.94
	*Silurus glanis*	29.87	8.49	NA

## Discussion

The results of our study provide valuable insights into the potential predation impact and competition of the introduced European perch *Perca fluviatilis* on the fish community of ﻿İznik Lake. Our findings support our hypothesis that the impacts of perch introductions would vary based on the source population and manifest as differences in predation impact and competition with other apex predators^38^. These differences in dietary preferences and trophic positions confirm that the source population of perch influences their ecological impacts. Furthermore, our results also confirm that the introduction of *P. fluviatilis* could lead to competition with other apex predators, such as *Silurus glanis,* as the overlap analysis and trophic position calculations revealed the potential for competition between these species, highlighting the potential disruption of predator–prey dynamics and the need for considering the consequences of introductions in freshwater ecosystems.

### Predation impact based on the mixing models

Stable isotope mixing models revealed important information about potential dietary impacts of introduced *P. fluviatilis* on the fish community of ﻿İznik Lake. The models indicated that if perch were introduced to the lake, they would primarily prey upon *Vimba vimba* and *Rutilus rutilus*, with *R. rutilus* being consumed to a lesser extent by perch from reservoirs, thereby confirming the results of previous work^39,40^. These findings can be attributed to several ecological factors, both species being likely preferred prey for perch due to their ecological traits and availability^41^. Both species are known to occupy lower trophic levels within the fish community^42^, feeding on detritus, benthic invertebrates, and zooplankton^43,44^. These prey items provide an energy-rich food source for perch. *Vimba vimba* and *R. rutilus* are commonly found in the shallow areas of ﻿İznik Lake, where perch are known to be efficient predators due to their hunting strategies and adaptability to different habitats^45^. It should however be acknowledged that *P. fluviatilis* prefers slightly deeper zones compared to *R. rutilus*, meaning that their realized spatial overlap will depend on oxygen availability^46^. On the other hand, other fish species in the community were deemed unlikely to be consumed by perch. This could be attributed to a combination of factors, including differences in habitat preferences, feeding behavior, and size^47,48^. Some species, such as *Gambusia holbrooki*, *Capoeta tinca*, and *Salaria fluviatilis*, occupy different ecological niches or exhibit specific adaptations that reduce their vulnerability to predation by perch^49–52^. It is important to note that gobiid species like *Proterorhinus semilunaris*, are highly unlikely to occur in reservoirs located in remote areas established for irrigation purposes due to their incompatible habitat requirements^53^. On the other hand, *Atherina boyeri*, another translocated species, appears to be an important component of perch diet in both reservoirs and natural lakes, as evidenced by reports of perch preying on *A. boyeri* in Seyitler reservoir^54^. Additionally, larger species like *Cyprinus carpio* or *Silurus glanis* (this latter also being protected by its pectoral spines^55^) may exceed the preferred prey size range of perch, making them less suitable prey^26^. While we acknowledge that we only considered adult individuals as prey, unable to assess the potential impacts of perch on the young-of-the-year populations^56^, our findings highlight the potential impact of perch introductions on the prey populations in ﻿İznik Lake, with *V. vimba* and *R. rutilus* being particularly susceptible to predation. The selective predation on these species could have cascading effects throughout the food web, altering the community structure and ecosystem dynamics^57^.

### Competition with *Silurus glanis* for the position of apex predator

Our study identified the European catfish *S. glanis* as the sole apex predator in ﻿İznik Lake, with only *G. holbrooki* showing differentiation from the otherwise all-overlapping fish species. *Silurus glanis* occupied the top trophic position in the ecosystem, therefore exerting top-down control over the fish community dynamics. However, projecting the isotopic niche of the two groups of *P. fluviatilis* indicated a theoretical overlap with the *S. glanis* population, suggesting a potential shift in the apex predator status and competition for feeding resources within the ecosystem^58,59^.

Such competition for limited food resources among top predators can have profound ecological implications, potentially leading to changes in the community structure and dynamics^60,61^. The overlap potential and niche width analysis further supported the likelihood of competition between *P. fluviatilis* and *S. glanis*. The considerable overlapping potential between the two species in terms of standard ellipse areas (SEAb) suggests that they may have similar dietary preferences and utilize similar feeding habitats within the lake. This implies that the introduction of *P. fluviatilis* could potentially disrupt the existing trophic interactions and resource partitioning, leading to increased competition for prey resources and potential changes in the distribution and abundance of both species^59^. When examining congruence, it is however important to take into account not only the contrasting sizes of *P. fluviatilis* and *S. glanis*, but also the varying sizes of their potential prey. *Silurus glanis*, in addition to actively hunting throughout the water column, also consumes decaying matter such as dead fish found on the lake bottom^58^. On the other hand, due to limitations in their mouth size, *P. fluviatilis* is likely to primarily target smaller prey fish, including young-of-the-year and juveniles, which subsequently prevents them from growing and becoming prey for *S. glanis*.

These findings highlight the importance of considering the potential ecological consequences of *P. fluviatilis* introductions, especially in relation to the existing apex predator, *S. glanis*, in the ecosystem. The potential competition between these two top predators raises concerns about the stability and functioning of the fish community in ﻿İznik Lake. Understanding the ecological implications of such interactions is crucial for making informed decisions regarding the introduction of non-native species and managing the conservation and sustainability of aquatic ecosystems.

### Caveats

One of the challenges in our study was standardization of the isotope data from the target community and the different perch populations, as the baseline organisms differed. Considering that even standardization with baseline organisms can introduce a certain bias based on differences in baseline organisms, which may locally differ even within ecosystems (see Ref.^35^ for a detailed discussion), we employed a novel approach by superimposing raw stable isotopes data from multiple perch populations into the recipient community stable isotopes biplot, assuming that the focal species niche would position itself somewhere in between the range of the superimposed data^35,61^. While this approach may introduce some uncertainties, it provides a rough estimation of the predation impact and competition dynamics, and—due to data availability limitations—such a situation is often the best compromise^35^. Concomitantly, we found similar results between *P. fluviatilis* originating from lakes and reservoirs, underlining the reliability of our results.

In light of the intricate web of interactions within the fish community, we acknowledge the critical role played by size guilds and body characteristics in shaping the potential impacts of *P. fluviatilis* introductions. Species-specific attributes, such as body depth and size can prevent perch predation, as exemplified by the deep-bodied morph observed in *C. gibelio*^54^. This adaptation provides a degree of protection even in the presence of larger perch. Conversely, smaller-sized species and younger individuals, as indicated in Table 3, may face increased vulnerability to predation. While our study primarily focuses on average food niche dynamics, we recognize the need for a more nuanced consideration of these factors in future studies by investigating empirical evidence regarding the post-introduction trends in these vulnerable species, seeking to provide a more comprehensive understanding of the ecological consequences of perch introduction.

**Table 3:** Status (Inv= invasive, Trans= translocated, Nat= native), number of samples (n), average and standard deviation (SD) of δ^15^N and δ^13^C, mean total length (TL), and weight (W) of fish species and other groups collected from İznik Lake.

Species	Status	n	Average δ^15^N	SD δ^15^N	Average δ^13^C	SD δ^13^C	Mean TL [mm]	Mean W [g]
*Atherina boyeri*	Trans	10	9.19	0.35	– 21.44	0.29	93.10	5.15
*Capoeta tinca*	Nat	1	15.72	NA	– 19.88	NA	256.00	162.00
*Carassius gibelio*	Inv	10	7.03	0.56	– 21.12	1.30	137.80	44.35
*Cyprinus carpio*	Nat	4	7.62	1.72	– 20.06	0.97	104.00	17.63
*Gambusia holbrooki*	Inv	10	10.65	0.85	– 17.95	0.77	40.60	0.90
*Knipowitschia caucasica*	Nat	7	8.46	1.01	– 20.18	1.25	27.86	0.19
*Proterorhinus semilunaris*	Nat	12	7.10	1.61	– 21.54	1.35	42.55	1.03
*Rutilus frisii*	Nat	10	9.44	0.91	– 20.85	2.11	218.40	117.45
*Rutilus rutilus*	Nat	10	9.25	0.33	– 22.90	0.62	143.20	27.80
*Salariopsis fluviatilis*	Nat	20	7.93	1.38	– 19.98	1.36	30.33	0.27
*Silurus glanis*	Nat	10	12.60	3.70	– 24.65	2.35		
*Vimba vimba*	Nat	10	9.89	0.91	– 22.38	0.99	143.10	26.60
*Pelophylax ridibundus*	Nat	3	7.58	1.66	– 17.19	1.72		
Macrozoobenthos		1	5.25	NA	– 20.25	NA		
Phytoplankton		2	3.79	0.23	– 22.00	0.07		
Macrophytes		2	1.23	3.48	– 10.10	0.56		
Algae		1	2.09	NA	– 20.58	NA		
Detritus		3	5.63	4.65	– 16.60	4.73		
Zooplankton		2	5.63	0.43	– 24.27	1.07		

### Management implications: steps to be taken for invasive perch

By utilizing stable isotope analyses and considering the ethological differences in behavior between perch source populations, we were able to gain valuable insights into the potential ecological consequences or repercussions of potential perch introductions. Our approach, although not perfect, contributes to our understanding of the potential impacts of invasive species introductions and serves as a starting point for further research and management decisions.

*Perca fluviatilis* has been recognized as an invasive species with the potential to disrupt native biodiversity^24,62^. Considering the aggressive feeding behavior and competitive advantage of perch^63^, proactive measures should be taken to mitigate the risk of perch introductions before they occur. The recent surge in popularity of perch as a target species for angling has led to widespread introductions, particularly in reservoirs^54^. The active involvement and support of local and national angling clubs in this initiative suggest that the spread of perch may occur rapidly and effectively in the coming years. Being a large natural lake, it is possible that the results obtained from *P. fluviatilis* originating from lakes may be behaviourally—and possibly trophically—more similar than individuals from reservoirs^54^. It is however possible that observed behavioral differences (i.e. higher aggressiveness in reservoirs; pers. communication Necati Ayvaz) could be due to the presence of schooling prey^64^, which could lead to different impacts based on the origin of the source population – in the case *P. fluviatilis* was to be introduced into ﻿İznik Lake. Additionally, recent research indicates that perch may become a preferred prey species for European catfish, potentially leading to a mesopredator status and introducing a new layer of complexity to the ecological dynamics, with *S. glanis* functioning as natural control agent^27,28^. This relationship underscores the need for a comprehensive assessment of species interactions in the context of perch introductions. Hence, our study highlights the need for targeted strategies for invasive species management, focusing on the preservation of native communities and the ecological integrity of freshwater ecosystems. Implementing strict regulations on the movement and introduction of invasive species, along with comprehensive monitoring.

The findings from our study will have important implications for ecosystem managers, stakeholders, and policymakers, highlighting the significance of proactive measures in mitigating biological invasions before they occur. By understanding the potential impacts of invasive species introductions, we can develop targeted strategies for invasive species management and prioritize the conservation of native communities and the ecological integrity of freshwater ecosystems.

## Methods

### Study sites

**Figure 4:**
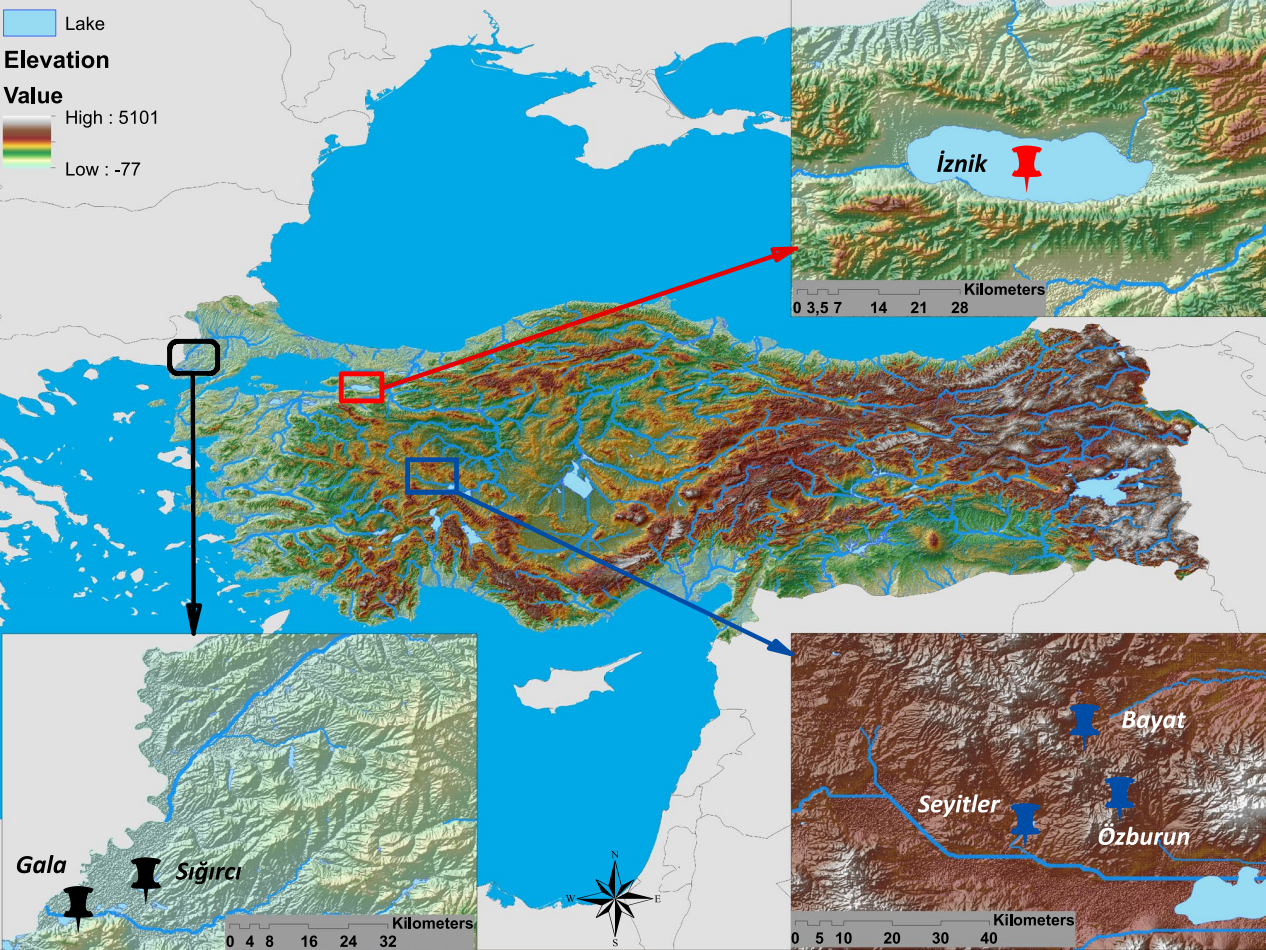
Sampling sites in western Anatolia, Turkey. The map was created with Arcgis 10.8.

İznik Lake is situated between the towns of Orhangazi and ﻿İznik, with coordinates ranging from 40° 23′ to 40° 30′ N and 29° 30′ to 29° 42′ E. It is located at 80 m a.s.l. and, with an area of 313 km^2^, is the largest water body in the Marmara Region and the fifth largest lake in Turkey (Fig. 4). This deep tectonic lake is fed by small streams, creeks, and groundwater. However, due to high agricultural activity in the surrounding areas and the discharge of wastewater from nearby residential areas, the lake has experienced an increase in nutrient concentrations^65^. Consequently, its trophic status has shifted from oligotrophic to mesotrophic in recent decades^38^. İznik Lake is home to more than 20 fish species, predominantly cyprinids^45^ (Table 3).

### Sample collection and laboratory processing

Sampling was conducted during autumn (October–November) of 2021. Fish were captured using multi-mesh gillnets consisting of 12 panels with varying mesh sizes ranging from 5 to 55 mm. Once captured, fish were transported to the laboratory in an ice water slurry. Samples of potential prey resources, including submerged macrophytes, algae, phytoplankton, zooplankton, macroinvertebrates (i.e. bivalves, gastropods), and detritus were collected from each sampling site, whenever available. Macroinvertebrates and macrophytes were collected with a grab and a scoop from the lake bottom and surface, while phytoplankton and zooplankton were collected with a plankton net (50 µm).

In total, twelve fish species, three individuals of the marsh frog *Pelophylax ridibundus* and other organisms (Table 3) were collected. For fish species, we used a caliper with an accuracy of 0.01 mm to measure the total length (TL). The weight (W) was measured using an electronic balance with an accuracy of 0.01 g. For all individuals of each fish species and frog, a sample of muscle tissue was extracted for stable isotopes analysis. The muscle tissue was thoroughly cleaned to remove any fat, skin, scales, and bones. Macrophyte samples were rinsed with tap water and, after removing insects and other organic materials. All samples were stored at − 20 °C for stable isotope analysis. Other organisms were processed as a whole, except for mollusks for which the shell was removed. The samples were placed in separate glass trays and dried for 48 h in an oven set at 60 °C. After drying, the samples were finely ground into a powder using an agate mortar and pestle. The combusted samples were subsequently analyzed using a Thermo Finnigan Delta Plus Advantagean isotope ratio mass spectrometer at the Biological and Chemical Research Centre in Warsaw, Poland. The isotope compositions were expressed as ‰ using the δ notation, calculated based on δ^13^C or δ^15^N = ((R_sample_/R_standard_) − 1) × 1000, where R represents the ^13^C:^12^C or ^15^N:^14^N ratios, and the standards were Vienna Pee Dee Belemnite for carbon and atmospheric N_2_ for nitrogen.

### Ethical approval for research involving human participants and/or animal

This article does not contain any studies with human participants performed or experiments conducted on animals by any of the authors. The legal permission for collecting fish was provided by the Republic of Türkiye Ministry of Agriculture and Forestry (E-67852565-140.03.03-1800883). All care and use of experimental animals were complied with animal welfare international/national laws, guidelines and policies. Some fish were obtained from the fisherman (Nazmi Tuna) with his consent.

### Obtaining perch isotope data

To investigate the potential effects of introducing *P. fluviatilis* on the fish community of ﻿İznik Lake, we collected perch δ^13^C and δ^15^N data from five water bodies in western Anatolia. Three of these water bodies are reservoirs (Seyitler, Bayat, and Ozburun reservoirs) established for mainly irrigation purposes, ranging in size from 0.05 to 4.5 km^2^, with average maximum depths of approximately 25 m. The remaining two study sites are natural lakes (Gala and Sigirci lakes) of similar sizes, around 6 km^2^, and primarily shallow, with depths ranging between 2 and 6 m. The reservoirs have a limited fish fauna, with perch as the only predator species, along with a few other native and non-native species. On the other hand, the two native lakes harbor a higher number of species, including several native predator species such as *Silurus glanis* and *Sander lucioperca*, in addition to perch.

### Statistical analyses

To calculate the trophic position (TP) for each species, we used a two-baseline model (baseline 1: detritus; baseline 2: phyto- and zooplankton^66^) implemented in the R package tRophicPosition, which uses Markov Chain Monte Carlo simulations^67^. Because species-specific trophic discrimination factors (TDFs) were not available for all species, we generated values based on the available data using the R function *simulateTDF*^67^. We analyzed the stable isotope data of *P. fluviatilis* and observed differences between the signatures found in lakes versus reservoirs. This led us to consider the potential effects of both lake and reservoir environments on the fish fauna of ﻿İznik Lake, individually assessing their impacts on the fish community.

The application of dual plot graphs for δ^15^N versus δ^13^C of consumer tissues and food sources enabled the determination of probable prey sources and combinations of prey contributing to the diet of predators^68^. To estimate how sampled species would contribute to the diet of possibly introduced *P. fluviatilis* from reservoirs and lakes, stable isotope mixing models were applied using the R package simmr^69^. These models were conducted for the two groups of *P. fluviatilis* (native specimens from lakes and introduced individuals from reservoirs) possibly introduced to ﻿İznik Lake. As potential prey items, we considered all fish species except *Silurus glanis*, which was, based on its apex position and size, considered as unsuitable^70,71^. Results of the mixing models are presented as a posteriori distribution for the proportion of each prey item in the diet.

The standard ellipse areas corresponding to the Bayesian 95% standard ellipse area (SEAb) and the area corresponding to 40% of central data points (SEAc) were calculated using the R package SIBER^72^ for both groups of *P. fluviatilis* and *S. glanis*^72^. Using the same package, we computed the degree of isotopic niche overlap (ranging from 0: no overlap, to 1: complete overlap) as a proxy for trophic competition among populations^72^. Finally, the directional pairwise probability of the two groups of *P. fluviatilis* and *S. glanis* to overlap their niche was estimated using the R package nicheROVER, which applies a Monte Carlo estimation (chain length: 10,000 steps) on the potential overlap, thus computing the directional pairwise probability of the niche of one species (not either species) overlapping onto the niche of another to further assess the direction of the potential trophic competition^32,67^. 

### Supplementary Information

Supplementary Information.

## Data Availability

The data used in this study can be obtained from Ali Serhan Tarkan upon reasonable request by contacting the lead author.

## References

[1] Poulin R, Paterson RA, Townsend CR, Tompkins DM, Kelly DW (2011). Biological invasions and the dynamics of endemic diseases in freshwater ecosystems. Freshw. Biol..

[2] Cassini MH (2020). A review of the critics of invasion biology. Biol. Rev..

[3] Gozlan RE, Britton JR, Cowx I, Copp GH (2010). Current knowledge on non-native freshwater fish introductions. J. Fish Biol..

[4] Haubrock PJ (2022). Invasion impacts and dynamics of a European-wide introduced species. Glob. Change Biol..

[5] Blackburn TM (2011). A proposed unified framework for biological invasions. Trends Ecol. Evol..

[6] Warren RJ (2019). Release from intraspecific competition promotes dominance of a non-native invader. Biol. Invasions.

[7] Marr SM (2013). A global assessment of freshwater fish introductions in mediterranean-climate regions. Hydrobiologia.

[8] Fricke R, Bilecenoğlu M, Sarı HM (2007). Annotated checklist of fish and lamprey species (Gnathostomata and Petromyzontomorphi) of Turkey, including a Red List of threatened and declining species. Stuttg. Beitr. Naturkd..

[9] Tarkan AS, Marr SM, Ekmekçi FG (2015). Non-native and translocated freshwater fish. FISHMED Fish. Mediterr. Environ..

[10] Tarkan AS (2017). Identification of potentially invasive freshwater fishes, including translocated species, in Turkey using the aquatic species invasiveness screening kit (AS-ISK). Int. Rev. Hydrobiol..

[11] Çiçek E, Sungur S, Fricke R (2020). Freshwater lampreys and fishes of Turkey; a revised and updated annotated checklist 2020. Zootaxa.

[12] Copp GH (2005). To be, or not to be, a non-native freshwater fish?. J. Appl. Ichthyol..

[13] Hodder KH, Bullock JM (1997). Translocation of native species in the UK: Implications for biodiversity. J. Appl. Ecol..

[14] Glamuzina B (2017). Comparison of taxon-specific and taxon-generic risk screening tools for identifying potentially invasive non-native fishes in the River Neretva catchment (Bosnia & Herzegovina and Croatia). River Res. Appl..

[15] Piria M (2016). Risk screening of non-native freshwater fishes in Croatia and Slovenia using FISK (Fish Invasiveness Screening Kit). Fish. Manag. Ecol..

[16] Vander Zanden MJ, Olden JD (2008). A management framework for preventing the secondary spread of aquatic invasive species. Can. J. Fish. Aquat. Sci..

[17] Tricarico E (2012). A review on pathways and drivers of use regarding non-native freshwater fish introductions in the Mediterranean region. Fish. Manag. Ecol..

[18] Strayer DL (2010). Alien species in fresh waters: Ecological effects, interactions with other stressors, and prospects for the future. Freshw. Biol..

[19] Simberloff D (2013). Impacts of biological invasions: What’s what and the way forward. Trends Ecol. Evol..

[20] Haubrock PJ (2019). Predicting the effects of reintroducing a native predator (European eel,* Anguilla anguilla*) into a freshwater community dominated by alien species using a multidisciplinary approach. Manag. Biol. Invasions.

[21] Sih A (2010). Predator–prey naïveté, antipredator behavior, and the ecology of predator invasions. Oikos.

[22] Balzani P, Gozlan RE, Haubrock PJ (2020). Overlapping niches between two co-occurring invasive fish: The topmouth gudgeon *Pseudorasbora*
*parva* and the common bleak *Alburnus*
*alburnus*. J. Fish Biol..

[23] Elvira B, Almodóvar A (2001). Freshwater fish introductions in Spain: Facts and figures at the beginning of the 21st century. J. Fish Biol..

[24] Bakiu R (2022). Invasiveness assessment of European perch (*Perca*
*fluviatilis*), pike-perch (*Sander*
*lucioperca*) and northern pike (*Esox*
*lucius*) in Albanian freshwater ecosystems by using the aquatic species invasiveness screening kit (AS-ISK). Stud. Mar..

[25] Freyhof, J. & Kottelat, M. *Perca fluviatilis*. The IUCN Red List of Threatened Species 2008: e.T16580A6135168. 10.2305/IUCN.UK.2008.RLTS.T16580A6135168.en. (Accessed 10 June 2023) (2008).

[26] Adámek Z, Musil J, Sukop I (2004). Diet composition and selectivity in O+ perch (*Perca*
*fluviatilis* L.) and its competition with adult fish and carp (*Cyprinus*
*carpio* L.) stock in pond culture. Agric. Conspec. Sci..

[27] Vejřík L (2017). European catfish (*Silurus*
*glanis*) as a freshwater apex predator drives ecosystem via its diet adaptability. Sci. Rep..

[28] Šmejkal M, Ricard D, Sajdlová Z (2018). Can species-specific prey responses to chemical cues explain prey susceptibility to predation?. Ecol. Evol..

[29] Hempel M, Neukamm R, Thiel R (2016). Effects of introduced round goby (*Neogobius*
*melanostomus*) on diet composition and growth of zander (*Sander*
*lucioperca*), a main predator in European brackish waters. Aquat. Invasions.

[30] Cucherousset J (2012). Using stable isotope analyses to determine the ecological effects of non-native fishes. Fish. Manag. Ecol..

[31] Trueman CN, MacKenzie KM, Palmer MR (2012). Identifying migrations in marine fishes through stable-isotope analysis. J. Fish Biol..

[32] Haubrock PJ (2021). Spatio-temporal niche plasticity of a freshwater invader as a harbinger of impact variability. Sci. Total Environ..

[33] Boecklen WJ, Yarnes CT, Cook BA, James AC (2011). On the use of stable isotopes in trophic ecology. Annu. Rev. Ecol. Evol. Syst..

[34] Post DM (2002). Using stable isotopes to estimate trophic position: Models, methods, and assumptions. Ecology.

[35] Balzani P, Haubrock PJ (2022). Expanding the invasion toolbox: Including stable isotope analysis in risk assessment. NeoBiota.

[36] Top Karakuş N, Tarkan AS (2022). Does non-native pumpkinseed *Lepomis*
*gibbosus* affect endemic algae-scraping *Capoeta*
*aydinensis* in case of introduction to a small stream? An ex-situ growth experiment. Ecol. Freshw. Fish.

[37] Emiroğlu Ö, Aksu S, Başkurt S, Britton JR, Tarkan AS (2023). Predicting how climate change and globally invasive piscivorous fishes will interact to threaten populations of endemic fishes in a freshwater biodiversity hotspot. Biol. Invasions.

[38] Jacobson P, Bergström U, Eklöf J (2019). Size-dependent diet composition and feeding of Eurasian perch (*Perca*
*fluviatilis*) and northern pike (*Esox*
*lucius*) in the Baltic Sea. Boreal Environ. Res..

[39] Persson L, Eklov P (1995). Prey refuges affecting interactions between piscivorous perch and juvenile perch and roach. Ecology.

[40] Persson L, De Roos AM, Byström P (2007). State-dependent invasion windows for prey in size-structured predator-prey systems: Whole lake experiments. J. Anim. Ecol..

[41] Dörner H, Wagner A (2003). Size-dependent predator-prey relationships between perch and their fish prey. J. Fish Biol..

[42] Hayden B (2014). Trophic flexibility by roach *Rutilus*
*rutilus* in novel habitats facilitates rapid growth and invasion success. J. Fish Biol..

[43] Đikanović, V., Čanak Atlagić, J., Zorić, K., Ilić, M. & Skorić, S. The diet of 22 fish species in the Belgrade sector of the Danube River. In Abstracts of the 3rd CESAMIR, Central Europen Symposium for Aquatic Macroinvertebrates Research, 99–99. Department of Invertebrate Zoology & Hydrobiology University of Łódź (2018).

[44] Ostaszewska T, Dabrowski K, Hliwa P, Gomółka P, Kwasek K (2008). Nutritional regulation of intestine morphology in larval cyprinid fish, silver bream (*Vimba*
*vimba*). Aquac. Res..

[45] Özuluğ M, Altun Ö, Meriç N (2005). On the fish fauna of İznik Lake (Turkey). Turk. J. Zool..

[46] Prchalová M (2009). The effect of depth, distance from dam and habitat on spatial distribution of fish in an artificial reservoir. Ecol. Freshw. Fish.

[47] Truemper HA, Lauer TE (2005). Gape limitation and piscine prey size-selection by yellow perch in the extreme southern area of Lake Michigan, with emphasis on two exotic prey items. J. Fish Biol..

[48] Mihalitsis M, Bellwood DR (2017). A morphological and functional basis for maximum prey size in piscivorous fishes. PLoS One.

[49] Blanco-Garrido F, Clavero M, Prenda J (2009). Jarabugo (*Anaecypris*
*hispanica*) and freshwater blenny (*Salaria*
*fluviatilis*): Habitat preferences and relationship with exotic fish species in the middle Guadiana basin. Limnetica.

[50] Murphy CA, Grenouillet G, García-Berthou E (2015). Natural abiotic factors more than anthropogenic perturbation shape the invasion of Eastern Mosquito fish (*Gambusia*
*holbrooki*). Freshw. Sci..

[51] Kaya, C. Taxonomic revision of the species belong to genus *Capoeta* distributed in Turkey. PhD Thesis. Recep Tayyip Erdogan University, Institute of Science and Technology, 126 (2019).

[52] Kurtul I, Tarkan AS, Sarı HM, Britton JR (2022). Climatic and geographic variation as a driver of phenotypic divergence in reproductive characters and body sizes of invasive *Gambusia*
*holbrooki*. Aquat. Sci..

[53] Top N, Karakuş U, Tepeköy EG, Britton JR, Tarkan AS (2019). Plasticity in habitat preferences of two native Ponto-Caspian gobies, *Proterorhinus*
*semilunaris* and *Neogobius*
*fluviatilis*: Implications for invasive populations. Knowl. Manag. Aquat. Ecosyst..

[54] Tarkan AS (2023). Phenotypic responses to piscivory in invasive gibel carp populations. Aquat. Sci..

[55] Kalish-Achrai N, Monsonego-Ornan E, Shahar R (2017). Structure, composition, mechanics and growth of spines of the dorsal fin of blue tilapia *Oreochromis aureus* and common carp *Cyprinus*
*carpio*. J. Fish Biol..

[56] Westrelin S, Balzani P, Haubrock PJ, Santoul F (2023). Interannual variability in the trophic niche of young-of-year fish belonging to four piscivorous species coexisting in a natural lake. Freshw. Biol..

[57] Diehl S (1992). Fish predation and benthic community structure: The role of omnivory and habitat complexity. Ecology.

[58] Copp GH (2009). Voracious invader or benign feline? A review of the environmental biology of European catfish *Silurus*
*glanis* in its native and introduced ranges. Fish Fish..

[59] Sicuro B, Tarantola M, Valle E (2016). Italian aquaculture and the diffusion of alien species: Costs and benefits. Aquac. Res..

[60] Guillerault N (2015). Does the non-native European catfish *Silurus*
*glanis* threaten French river fish populations?. Freshw. Biol..

[61] Haubrock PJ, Azzini M, Balzani P, Inghilesi AF, Tricarico E (2020). When alien catfish meet—Resource overlap between the North American *Ictalurus*
*punctatus* and immature European *Silurus glanis* in the Arno River (Italy). Ecol. Freshw. Fish..

[62] UK, C. *Perca fluviatilis* (Linnaeus, 1758), perch.[invasive species]. *Perca fluviatilis* (Linnaeus, 1758), perch. [invasive species], (AQB ISC record) (2014).

[63] Beeck, P. The early piscivory of European perch (*Perca fluviatilis*)—A neglected phenomenon with notable consequences for the population structure and fish community in lake ecosystems. PhD Thesis, University of Cologne, 115 (2003).

[64] Neill S, Cullen JM (1974). Experiments on whether schooling by their prey affects the hunting behaviour of cephalopods and fish predators. J. Zool..

[65] Akçaalan R, Mazur-Marzec H, Zalewska A, Albay M (2009). Phenotypic and toxicological characterization of toxic *Nodularia*
*spumigena* from a freshwater lake in Turkey. Harmful Algae.

[66] Haubrock PJ, Balzani P, Britton JR, Haase P (2020). Using stable isotopes to analyse extinction risks and reintroduction opportunities of native species in invaded ecosystems. Sci. Rep..

[67] Swanson HK (2015). A new probabilistic method for quantifying n-dimensional ecological niches and niche overlap. Ecology.

[68] Phillips DL, Gregg JW (2003). Source partitioning using stable isotopes: Coping with too many sources. Oecologia.

[69] Parnell A. & Inger R. Simmr: A stable isotope mixing model. R package version 0.3. https://cran.r-project.org/web/packages/simmr/index.html (2016).

[70] Boulêtreau S, Santoul F (2016). The end of the mythical giant catfish. Ecosphere.

[71] Bergström K (2022). Exceptional longevity in northern peripheral populations of Wels catfish (*Silurus*
*glanis*). Sci. Rep..

[72] Jackson AL, Inger R, Parnell AC, Bearhop S (2011). Comparing isotopic niche widths among and within communities: SIBER-stable isotope Bayesian ellipses in R. J. Anim. Ecol..

