# Supplementary material for: Predicting the potential implications of perch (Perca fluviatilis) introductions to a biodiversity-rich lake using stable isotope analysis
Source: Sci Rep. 2023 Oct 17;13:17635. doi: 10.1038/s41598-023-44865-2 (PMC10582113; doi:10.1038/s41598-023-44865-2)

**Supplementary Material: Predicting the Potential Implications of Perch (*Perca fluviatilis*) Introductions to a Biodiversity-rich Lake Using Stable Isotope Analysis**

**Table S1.** Mean and standard deviation (SD) of carbon (δ^13^C ) and nitrogen (δ^15^N) isotope values of species present in Iznik Lake.

| **Species** | **Mean dC** | **SD dC2** | **Mean dN** | **SD dN2** |
| --- | --- | --- | --- | --- |
| *Atherina boyeri* | -21.4379 | 0.2898 | 9.1897 | 0.3550 |
| *Capoeta tinca* | -19.8780 | 0.1000 | 15.7240 | 0.1000 |
| *Carassius gibelio* | -21.1203 | 1.2983 | 7.0296 | 0.5608 |
| *Cyprinus carpio* | -20.0555 | 0.9747 | 7.6243 | 1.7223 |
| *Pelophylax ridibundus* | -17.1853 | 1.7153 | 7.5830 | 1.6616 |
| *Gambusia holbrooki* | -17.9503 | 0.7718 | 10.6526 | 0.8471 |
| *Knipowitschia caucasica* | -20.1753 | 1.2498 | 8.4581 | 1.0104 |
| *Proterorhinus semilunaris* | -21.5414 | 1.3486 | 7.1018 | 1.6147 |
| *Rutilus frisii* | -20.8463 | 2.1057 | 9.4432 | 0.9052 |
| *Rutilus rutilus* | -22.9010 | 0.6248 | 9.2464 | 0.3314 |
| *Salariopsis fluviatilis* | -19.9765 | 1.3570 | 7.9310 | 1.3839 |
| *Silurus glanis* | -24.6507 | 2.3463 | 12.5976 | 3.6978 |
| *Vimba vimba* | -22.3802 | 0.9935 | 9.8901 | 0.9058 |

**Fig. S1.** Carbon (δ^13^C) and nitrogen (δ^15^N) stable isotopes biplot of translocated and native perch (*Perca fluviatilis*) present in study sites and putative prey species in Iznik Lake.


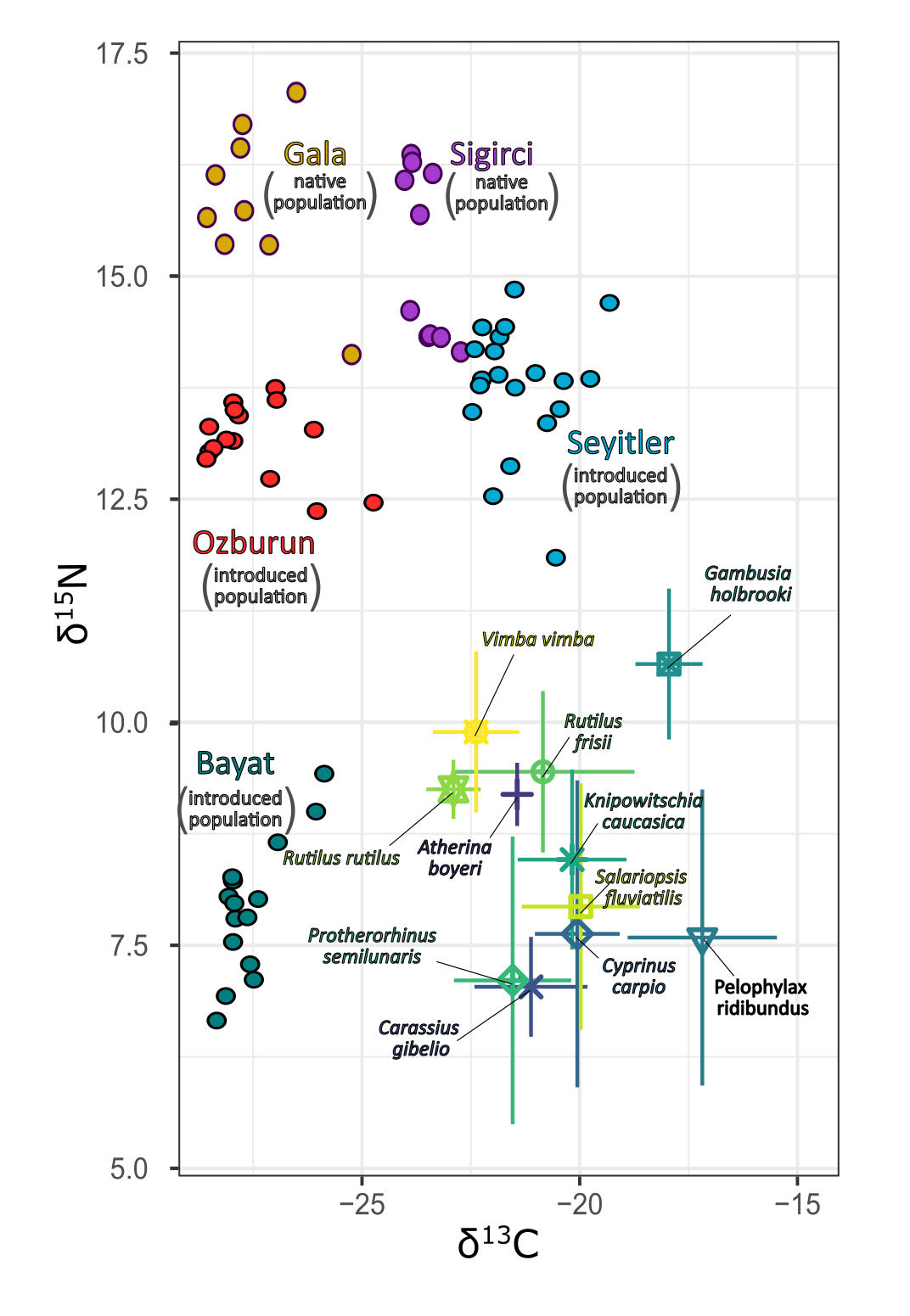


**Fig. S2.** Mixing models results for translocated and native perch (*Perca fluviatilis*) in the different sites and prey species in Iznik Lake as potentially available prey.


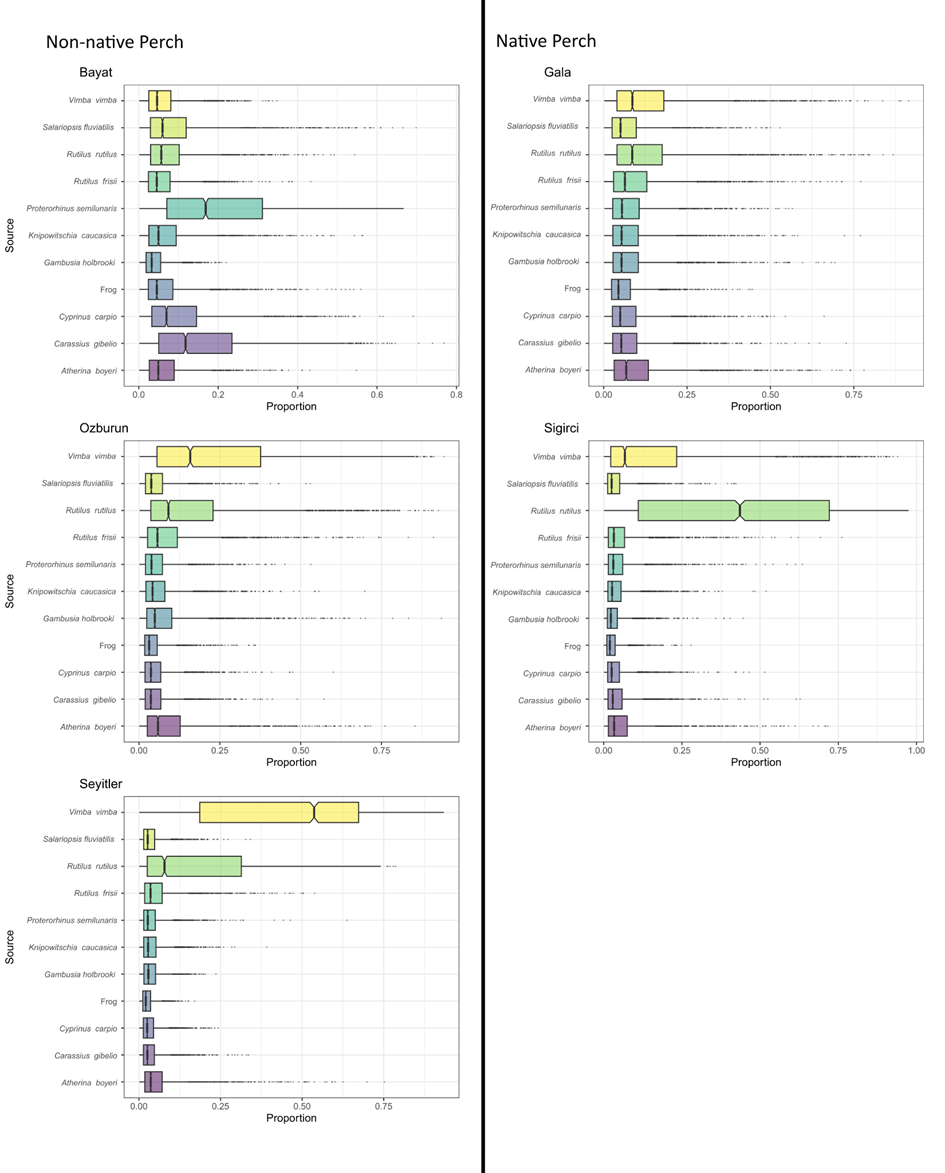

Supplement: Supplementary file 1 — Supplementary Information. [file 41598_2023_44865_MOESM1_ESM.docx]
